# Evaluation of the performance of the National Tuberculosis Program of Liberia during the 2014–2015 Ebola outbreak

**DOI:** 10.1186/s12889-019-7574-7

**Published:** 2019-09-04

**Authors:** Kassaye Tekie Desta, Dedeh Barr Kessely, Jerry G. Daboi

**Affiliations:** 1 Clinton Health Access Initiative, Monrovia, Liberia; 2 Atlantic Med System Consulting, Monrovia, Liberia; 3 National Leprosy and Tuberculosis Control Program of, Monrovia, Liberia; 4 Ministry of Health of Liberia National Diagnostic Unit, Monrovia, Liberia

**Keywords:** Ebola, Outbreak, TB, Treatment outcome, Case detection, TB/HIV

## Abstract

**Background:**

Liberia is among the three west African countries which were crippled by the Ebola Virus Disease (EVD) outbreak of 2014. One of the programs which was affected by the EVD outbreak was the National Leprosy and Tuberculosis Control Program (NLTCP). Determining the magnitude of the impact of EVD on the NLTCP performance is crucial in restoring the service and in devising effective post EVD strategies. The purpose of the study was to analyse the impact of EVD outbreak on the performance of the NLTCP of the Ministry of Health (MOH) OF Liberia.

**Methods:**

A cross sectional study design was conducted in 2016 using both quantitative and qualitative methods. Quantitative data was used for the Tuberculosis (TB) program evaluation before EVD (2012–2013) and during EVD (2014–2015). Qualitative data was used to complement the data obtained for the quantitative study. Descriptive statistical analyses of quantitative data were conducted using Microsoft Excel.

**Results:**

Notified TB cases of all forms decreased from 7822 in 2013 to 4763 and 6118 in 2014 and 2015 respectively. The number increased to 7180 and 7728 in 2016 and 2017 respectively. The TB treatment success rate was 71 and 61% in 2014 and 2015 respectively compared to the 83% in 2013. The treatment success rate was 77% in 2016. The loss to follow up (LTFU) was as high as 47% in some regions which were highly affected by the EVD outbreak. The national average LTFU was 5–10% in 2012–2013 and 16 and 21% in 2014 and 2015 respectively. The percentage of TB patients with known HIV result decreased from 75% in 2013 to 74 and 42% in 2014 and 2015 respectively. TB culture and drug susceptibility testing service was interrupted throughout the outbreak. The results of the focal group discussions and interviews conducted in our study also indicated that the TB case finding and the TB treatment outcome was significantly affected by the EVD outbreak.

**Conclusion:**

Notified TB cases and treatment outcome was significantly affected by the EVD outbreak which occurred in 2014 and 2015 in Liberia. Effective restoration strategies should be developed in order to improve the TB case finding and treatment outcome.

## Background

TB is a major public health problem worldwide. It affects the health of approximately 10 million people each year and is one of the top ten causes of death worldwide. TB is the ninth leading cause of death worldwide exceeding death from HIV/AIDS [1]. According to the World Health Organization (WHO) report of 2017; the TB incidence in Liberia for the year 2016 was 308/100,000 population compared to 242/100,000 in 2000 [2]. Liberia is among the high TB burden countries in Sub-Saharan Africa. The NLTCP of Liberia was well functioning starting from its establishment in 1989 under the Department of Preventive Health Services of the Ministry of Health and Social Welfare with TB case detection rate of above 60% and TB treatment success rate of above 70% between the 1990 and 2000 [3, 4]. Although few cases of TB were detected between 2005 and 2007, an increase in notification was observed from 2008 to 2012 notably because of the expansion of the program through funding from the Global Fund. The TB case notification of all forms was, 3456,4514 and 4511 in 2005,2006 and 2007 respectively. The notification increased to 5007 and 8132 in 2008 and 2012 respectively.

In Liberia, the treatment success rate was 76% in 2009 [4, 5]. This figure was by far less than the national and global targets of 85% set by the Stop TB strategy of WHO [6]. Treatment success rate is the number of new smear-positive pulmonary TB cases registered in a specified period that were cured plus the number that completed treatment divided by total number of new smear-positive pulmonary TB cases registered in the same period. Treatment outcome includes treatment success rate, LTFU, death rate and transfer out [7].

The recent west Africa EVD outbreak was first detected in Guinea in March 2014 and spread to the neighbouring countries of Liberia and Sierra Leone. As the result of this deadly outbreak, 28,616 cases and 11,310 deaths were reported in West Africa. Among this, 11,000 cases and 4800 deaths were reported in Liberia [8]. EVD is a severe, zoonotic, filovirus infection and life-threatening disease which spreads through direct contact with body fluids of a sick person with EVD [9]. Direct contact with the deceased person’s body during funeral or burial preparation is another means of transmission for the virus [10]. The 2014 west Africa EVD outbreak was the largest and longest Ebola epidemic and the first to occur in three entire neighbouring countries. The pre-existing health system of the three countries was already fragile before the outbreak due to years of armed conflict and poor economic status. This was further aggravated by the outbreak as result of death of health workers, fear of infection, closure of health facilities, delayed response of international community and stock out of medical supplies [11]. The devastating EVD outbreak in the three countries impacted significantly on all sectors of the healthcare systems including the TB prevention and control programs [12]. The outbreak hampered the diagnosis and treatment of HIV, malaria, TB and other diseases. Outpatient attendance was decreased significantly for fear of infection at the health facilities. Border closures, disruption of transportation route and curfew made access to routine medical services difficult [13].

In Liberia, the EVD outbreak began in March 2014 and caused a devastating impact on the health system, the population at large and the Liberian economy. As result of the 14 years of civil war which was started in 1989, the health system was on recovery and ill equipped to effectively respond to the epidemic with the necessary health facilities. The infection prevention measures, laboratory infrastructure and health work force were not in a position to contain the outbreak [14]. As a result of the existing weak infection prevention measures, the EVD epidemic had a 30 times higher risk of infection amongst Liberian health workers as compared to the general population, with 372 health workers infected, and 180 health workers died as of 18th March 2015 [15]. In addition to the weak infection prevention measures, the pre-existing structural vulnerabilities that aggravated the outbreak included inadequate and poorly motivated staff, insufficient and unsuitable infrastructure and equipment, weak supply chain and poor quality of care. During the EVD outbreak patients were screened and treated for EVD according to the WHO guidelines. All patients including presumed and confirmed TB were screened for fever at entry to each health facility. Any patient presenting with unexplained hemorrhage, or with a fever or who had been in contact with a known Ebola case, or an ill or dead animal, was referred to the Ebola Treatment Center in Liberia [16, 17].

Even though the exact magnitude of the impact of the EVD outbreak on TB was not exactly determined in Liberia, the health facility closures, community distrust, fears and refusal of health workers to provide routine health services has critically impacted the health service delivery. As a result, there were disruptions in provision of routine services. These disruptions have affected the entire health services. One of the programs which was affected by the EVD outbreak was the NLTCP. This was due to the temporary closure of the health facilities, fear and stock out of medical supplies. The impact of EVD on the health sector in general and TB in particular was studied in Sierra Leone and Guinea but it was not well studied in Liberia. The study findings from both countries indicated a decline in TB case notification and TB treatment outcome [13, 18]. This study is the first of its type in Liberia to exhaustively analyze the impact of EVD on TB case finding and treatment outcome in Liberia. It attempted to address the challenges caused by EVD with the ultimate aim of improving the TB treatment outcome to reduce Liberia’s TB-related mortality and morbidity rates with advantages for the individual TB patients and their families during the EVD outbreak and post EVD health service recovery. The study also provided recommendations to the National TB program on how to improve the post EVD TB treatment outcome and TB case finding and to effectively control future outbreaks and keep the momentum towards achieving the End TB strategies set by WHO for 2035 [6, 19].

## Methods

### Study design

A cross sectional study design was used for the study conducted in 2016 using both quantitative and qualitative study methods. Quantitative research design was used for the TB program evaluation before EVD (2012–2013) and during EVD (2014–2015). Quantitative study design was used for the study on the impact of EVD on TB program treatment outcome and case finding using the data obtained from TB program. NLTCP treatment outcome and laboratory diagnosis (2009–2017) data was extracted from the Ministry of Health of Liberia health management information system, NLTCP of Liberia documents and reports using a standard self-developed and piloted checklist. The data extracted included TB case notification rate, TB treatment outcome, TB/HIV co-infection trends and TB treatment success rate.

The qualitative study included two Focus Group Discussions (FGDs) and one semi structured interview questionnaires. The purpose of conducting these interviews and FGDs was to understand the real experience of the participants in evaluating any impact of EVD on TB program performance. The findings of these qualitative data analysis are considered to supplement the findings of the quantitative study.

### Study setting

The study was conducted in Liberia. Liberia is located in West Africa and is bounded by Guinea in the north, Cote d‘Ivoire in the east, Sierra Leone in the west and the Atlantic Ocean in the south. The country has a population of 4.3 million. The study setting included the NLTCP of Liberia where the TB program performance data was obtained. Besides, TB annex hospital which is co-located with NLTCP in Monrovia were used as study setting for the qualitative data collection.

### Study participants, sampling and data collection

The impact of EVD on TB program performance was evaluated from TB program managers and staff perspective. The study participants were selected using purposive sampling. Participants included TB program managers, county health team officers, county diagnostic supervisors, laboratory heads, nurses and laboratory technicians working in TB hospital, pharmacist and administrative staff. The qualitative data collection methods included individual interviews and FGDs. Two FGDS each composed of 10 people were included in the qualitative part of the study. The FGDs were facilitated by a moderator and the FGDs discussions were captured on the FGD guide. In addition to this, semi-structured interview questionnaires were conducted by the principal investigator. The interview participants were program managers, county health team officers, different facility TB staffs and county diagnostic officers. Altogether, 30 respondents participated of the 35 who consented. All the interviews and FGDs were conducted in English; the national language of Liberia.

### Data analysis

Thematic content analyses were performed on the qualitative data based on the FGD guides and semi-structured interview guide. Content analysis was used as it enables a systematic coding of data by organizing the information into categories to discover patterns undetectable by merely reading the transcripts. Inductive categories of themes and sub-themes emerged from the transcripts were used to draw original and unbiased conclusions. Direct quotes from the group discussion participants were not reported as each quote is captured in the themes and sub-themes. For the quantitative data, descriptive statistical analyses were conducted using Microsoft Excel.

### Ethical considerations

A written permission letter for TB program data and to conduct the study was obtained from the National Leprosy and Tuberculosis program of Liberia. Prior to conducting the study, permission was obtained in the form of a clearance certificate from the National Ethics Board of Liberia. Written consent was obtained from the FGD and interview study participants. Confidentiality was ensured at all stages of the study as no identifying details were written on the questionnaires, check lists and FGDs to link anyone to the data obtained. No information was given to third party without the permission of the Ministry of Health of Liberia.

## Results

### Performance of the TB laboratory diagnosis during the EVD outbreak

**Fig. 1:**
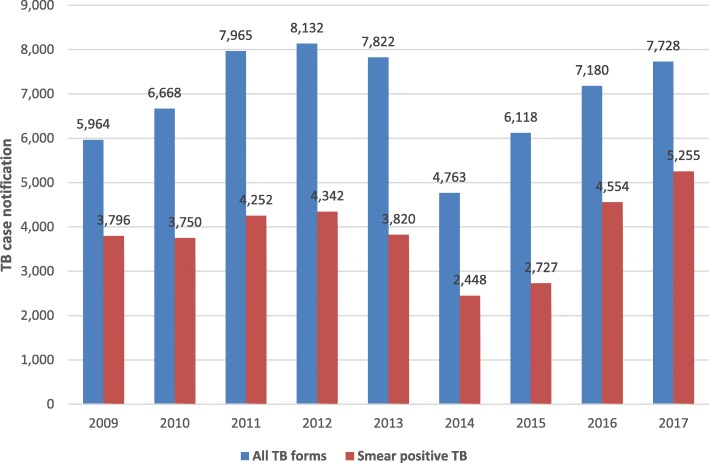
Tuberculosis case notification in Libera from 2009 to 2017. The graph in blue indicates notified TB cases of all forms and the graph in red indicates notified TB cases of smear positive per 100,000 population

**Fig. 2:**
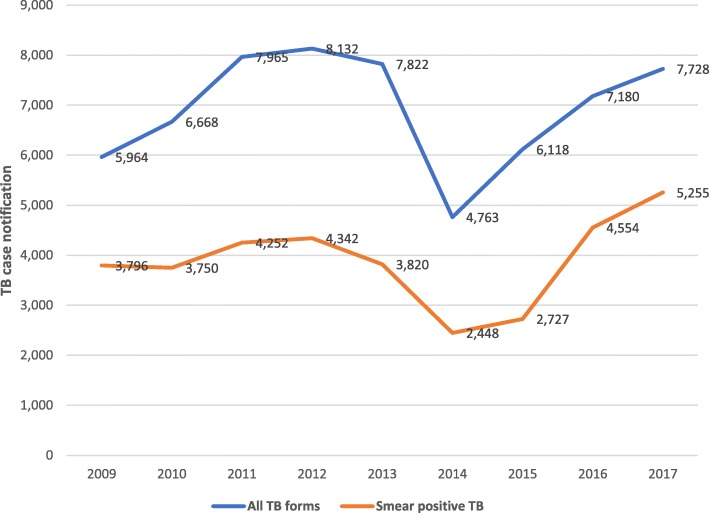
Tuberculosis case notification in Libera from 2009 to 2017. The line in blue indicates notified TB cases of all forms and the line in orange indicates notified TB cases of smear positive per 100,000 population

**Fig. 3:**
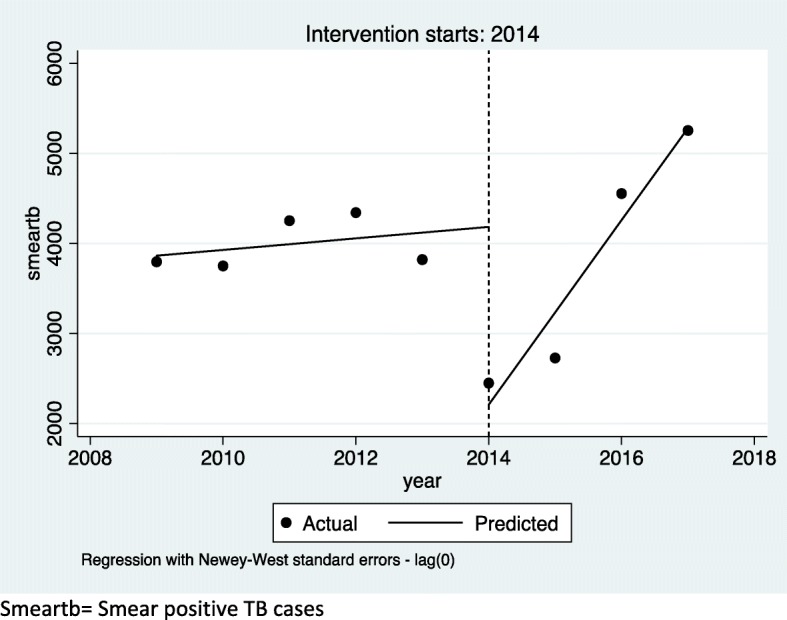
Regression with Newey -West adjusted standard errors. The line graph indicates the trend of TB positive smear cases before the EVD (2009–2013), during the EVD outbreak (2014–2015) and after the outbreak (2016–2017). Smeartb = Smear positive TB cases

The TB laboratory network in Liberia consists of one referral laboratory, one central laboratory, five regional laboratories and 167 peripheral microscopy service sites. The TB case notification of the NLTCP of Liberia before and during the 2014 EVD outbreak is shown below (Figs. 1 and 2). Notified smear positive cases of TB were 3820 in 2013 and dropped to 2448 during the 2014 heightened outbreak of EVD in Liberia. TB case notification had been increasing since 2011 but declined in 2014 and 2015.The TB case notification was in 2013 was 7822 and declined to 4763 and 6118 in 2014 and 2015 respectively. The number of TB cases notified increased to 7188 and 7728 in 2016 and 2017 respectively. The impact of the 2014 EVD outbreak on smear positive TB cases and on TB treatment success rate was analyzed using the interrupted time series analysis. Regression analysis with Newey -West adjusted standard errors was used to evaluate the impact of the 2014 EVD on smear positive TB cases. For smear positive cases, there was a significant change of level of the variable TB positive smear cases as well as in the trend of the number of cases of this variable (Fig. 3). This trend in notified TB cases clearly indicated that the decline in 2014 and 2015 was attributed to the EVD outbreak.

TB/HIV coinfection and collaboration efforts were also analyzed before and after the EVD outbreak to determine the impact of the outbreak on HIV testing of TB patients. In addition to this, the percentage of HIV ^+^ TB patients started or continued on Cotrimoxazole Preventive Therapy (CPT) and the percentage of HIV^+^ TB patients started or continued on Anti- Retroviral Therapy (ART) were compared before and after the EVD outbreak. The % of HIV ^+^ TB patients started or continued on CPT decreased during the outbreak but the % of HIV^+^ TB patients started or continued on ART increased during the outbreak as indicated below (Table 1).

**Table 1:** TB/HIV co-infection in Liberia (2011–2017)

TB/HIV Indicators	2011	2012	2013	2014	2015	2016	2017
Notified TB cases	7965	8132	7822	4763	6118	7180	7728
TB patients with known HIV test result	4355 (55%)	5661 (70%)	5899 (75%)	3567 (74%)	2577 (42%)	5313 (74%)	5410 (70%)
TB patients with positive HIV test	454 (10%)	772 (14%)	950 (16%)	538 (15%)	368 (14%)	829 (16%)	827 (15%)
HIV ^+^ TB patients started or continued on CPT	309 (68%)	324 (42%)	703 (74%)	301 (56%)	173 (47%)	NA*	NA*
HIV ^+^ TB patients started or continued on ART	45 (10%)	108 (14%)	152 (16%)	145 (27%)	92 (25%)	390 (47%)	347 (42%)

*NA: Data not available

Laboratory diagnostic supervisors’ perspectives of the impact of EVD on TB laboratory diagnosis.

This is a qualitative study where one FGD was conducted to get insight on the impact of EVD on TB laboratory performance from diagnostic supervisors’ perspective. A total of 10 county diagnostic supervisors participated in the FGD discussion. The work experiences of the participants were 6 to 20 years. There are 15 county diagnostic supervisors in Liberia who are assigned one per county. Of the 15 county diagnostic supervisors, 10 participated. The main themes that emerged from the FGD participants on the impact of EVD included TB laboratory diagnosis interruption as a result of facility closure for fear of EVD, priority was given to EVD and TB laboratory services were compromised. Besides, poor knowledge on the nature and transmission mechanism of EVD was another theme emerged during the FGD discussion. The sub-themes included work load as some laboratory technicians were repositioned for EVD response and lack of incentives and continuous stock out of laboratory reagents and supplies. FGDs were also asked to share their experience on the impact of EVD on Tuberculosis case finding. All the FGD participants mentioned that EVD affected the TB laboratory services in various ways. Most of the FGDs participants explained that the closure of the health facilities for fear of EVD crucially affected the TB services in their respective counties. The repurposing of resources to fight Ebola including logistics and man power was associated to the EVD impact on TB case finding by FGD participants. The lack of incentive to health workers including laboratory staff during the outbreak was argued by an FGD participant as the main cause of the negative impact on TB laboratory performance. Besides; the care given to the infected national staff was not intensive as those of infected expats and this was also discouraging as mentioned by an FGD participant.

### Impact of EVD on TB treatment outcome

**Fig. 4:**
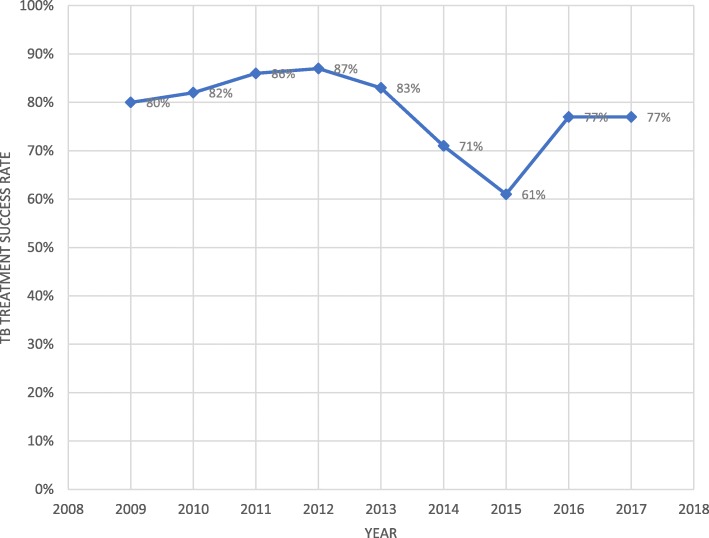
Tuberculosis treatment success rate (proportion of TB cases successfully treated; i.e. cured plus treatment completed among all TB cases notified) in Liberia from 2009 to 2017

**Fig. 5:**
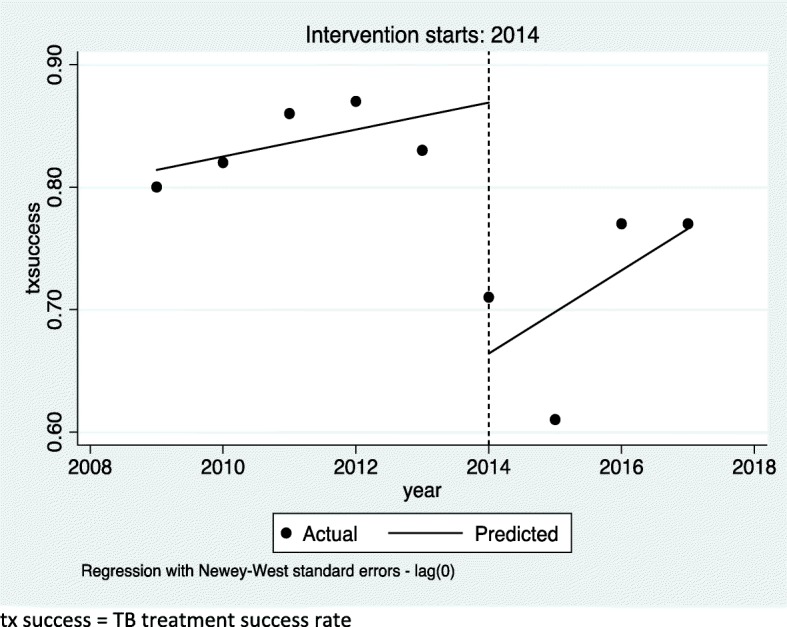
Regression with Newey -West adjusted standard errors. The line graph indicates the trend of TB treatment success rate before the EVD (2009–2013), during the EVD outbreak (2014–2015) and after the outbreak (2016–2017). tx success = TB treatment success rate

TB treatment success rate is considered as one of the of TB program treatment outcome indicators which help to evaluate the performance of national TB programs. Liberia made progress in maintaining a high treatment success rate above 80% in 2011 and 2012. In 2013, the treatment success rate was 83%. The treatment success rate of Liberia was also declined to 71% in 2014 and 61% in 2015 (Fig. 4). The TB treatment success rate increased to 77% in the year 2016 and 2017. Regression analysis using Newey -West adjusted standard errors showed no significant change both in the level of the variable treatment success rate and in the trend of this variable (Fig. 5). This indicated that Treatment success rate was not affected significantly during the 2014 EVD outbreak.

TB treatment outcome of cured rate, completed rate, failure rate, death rate and LTFU rate were also used as measures of the impact of EVD on TB treatment outcome. The most important cause of unfavourable outcome in Liberia after the EVD outbreak was the LTFU which was 7% in 2013 and became 16 and 21% during 2014 and 2015 respectively. The LTFU became 13% in 2016. The information obtained from the interview respondents and FGD participants of this study also indicated that the LTFU was as high as 47% in some regions of Liberia which were highly affected by the EVD outbreak in 2014. This was attributed to facility closures for fear of EVD and service interruption during the outbreak as indicated by the FGDs and interview respondents of our study. This had nevertheless by far increased from the national average LTFU rate of 9, 6, 4% in the 2010, 2011and 2012 respectively (Table 2).

**Table 2:** Trends of TB treatment out come in Liberia

TB treatment out come						
Year	Cured	Completed	Failed	Died	Transfer out	LTFU
2008	64%	15%	2%	4%	2%	13%
2009	57%	26%	1%	5%	1%	10%
2010	57%	25%	1%	5%	3%	9%
2011	64%	22%	1%	4%	3%	6%
2012	57%	30%	1%	4%	4%	4%
2013	62%	21%	1%	3%	6%	7%
2014	53%	18%	1%	7%	4%	16%
2015	50%	11%	4%	8%	6%	21%
2016	64%	13%	2%	6%	2%	13%

### NLTCP and TB hospital staff’s perspectives of the impact of EVD on TB treatment outcome

To get more insight of the impact of TB on EVD treatment outcome, one focal group discussion with 10 participants was included in the qualitative study. The composition of the FGD included two nurses, pharmacist, two medical doctors, two physician assistants, counselor nurse, laboratory technician and data clerk. The FGDs for this part of the study were selected purposively from each unit of the TB Annex hospital. The minimum years the FGD participants service in the TB referral hospital was 3. This was helpful to get in depth understanding of the program performance from the facility point of view and the impact of EVD on their facility performance. The main themes that emerged during the FGD discussion were interruption of TB facility service for fear of EVD, lack of logistics for TB service and lack of knowledge on the nature and transmission of the EVD and infection prevention mechanism. Stigma and denial were also mentioned as sub-themes. The other subthemes emerged were lack of government incentive for staff at high risk of infection, low salary, high staff turnover and poor service at the facility before the EVD outbreak.

FGD participants were also asked and discussed their experience on the impact of EVD outbreak on TB treatment outcome. Almost all participants explained that TB treatment outcome was highly affected by the EVD outbreak. They further explained that there was high rate of treatment interruption, failure and LTFU. FGD participants also indicated that EVD being a public health emergency during the outbreak, resources were prioritized to fight EVD and this also highly compromised the TB services.

The FGD participants provided the following recommendations on how to improve the post EVD TB treatment outcome in their facility as well as nationally.

- Improve the supply of TB drugs and laboratory reagents
- Initiate the interrupted GeneXpert and TB culture and drug susceptibility testing
- Build trust by ensuring safety of health workers and health facilities
- Improve the TB infection prevention from the lessons learnt from EVD Infection prevention practice
- Assess facility needs and filling their gap
- Involve the community to create awareness about TB so as to improve TB treatment adherence which was affected by EVD.

In addition to the two FGDs, Interview were conducted by the principal investigator using semi-structured interview guide. The interviews were conducted with TB program managers, county health team officers and county diagnostic officers as part of the qualitative study. The interview included 30 participants. The number of interview respondents who strongly agreed that EVD significantly impacted the TB laboratory diagnoses and treatment outcome of Liberia were 25(85%). Among the 30 interview respondents, 5(15%) associated the impact of EVD on TB laboratory diagnosis and treatment outcome to the existing poorly funded fragile health system before the EVD outbreak. The interview with the program managers at NLTCP also supported the later argument that there was some delay in the release of funding by Global fund. According to the interview respondents; the EVD outbreak also happened at the time where the TB program was writing the Global Fund to fight TB, AIDS and Malaria (GFTAM) round ten proposal. The delay from the GFTAM on the approval of the proposal was one impact. After this only extension grant was available throughout the EVD outbreak. The implementation of the proposed activities was delayed. The TB laboratory services including GeneXpert, Culture and drug susceptibility testing were interrupted during the outbreak and most of the data report indicated that TB smear was not done. Without a laboratory result, it was very difficult to meet the global case detection and treatment success rate of 70 and 85% respectively. The interview respondents further explained that the logistic and supply support from The MOH for the TB program was also critically affected by the outbreak and affected the program activities.

## Discussion

This is among the few studies conducted in the EVD affected west Africa countries to show the impact of EVD on the health service delivery. The findings of the study indicated that EVD negatively impacted the TB case finding and treatment outcome of Liberia. The first finding of our study was a sharp decline in the TB case notification of both smear positive TB cases and all forms of TB during the EVD outbreak. According to the FGD and interview participants of our study, the interruption of the routine TB sputum smear microscopy, TB culture, susceptibility testing and GeneXpert MTB/RIF during the outbreak were the main reason for the decline in TB case notification. Our findings were similar to the study findings of similar research conducted in Sierra Leone but different from the study findings in Guinea [18, 20]. A significant drop in case notification was observed in 2014 compared to 2015. In 2015, the health facilities were re-opened and the basic services were restored including the TB diagnosis and treatment. As result, there was an increase both in notified forms of all TB as well as notified cases of smear positive TB.

The findings of our study also indicated that the TB treatment outcome was significantly affected by the EVD outbreak. The treatment success rate of Liberia declined by EVD from 83% in 2013 to 71% in 2014 and 61% in 2015. This was lower than the Stop TB strategy target of 85% and by far lower than the End TB WHO target set after the Stop TB [6, 19]. This decline in treatment success rate was attributed to the high rate of LTFU of TB patients during the outbreak. The LTFU increased from 7% in 2013 to 16 and 21% in 2014 and 2015 respectively. The LTFU decreased to 13% in 2016 indicating an improvement in the performance of the TB program following the end of the outbreak. The TB treatment success rate also increased to 77% in 2016. Our study finding was similar to a study finding in Sierra Leonne [18]. The main reasons for the high LTFU deducted from the FGD participants and interview respondents of the study were closure of health facilities during the outbreak, decreased patients’ health facility visit for fear of EVD, treatment interruption, lack of laboratory reagents, poor knowledge of EVD and lack of standard infection prevention system at health facilities. The Ministry of Health of Liberia’s situational analysis of the post EVD health service indicated that more than 40% of health facilities across Liberia that previously provided TB diagnostic and treatment services were closed down between May and August 2014 [15]. A study conducted in West Africa also explained that the 2014–2015 Ebola outbreak in west Africa crippled the healthcare systems of the three countries, hampering diagnosis and treatment for endemic diseases such as malaria, HIV/AIDS and Tuberculosis [13, 21]. The death of health workers, closure of many health facilities and fear of EVD transmission affected the routine health service delivery including TB and HIV [22, 23]. During the EVD outbreak the percentage of TB patients who were tested for HIV declined to 3567(74%) and 2577(42%) in 2014 and 2015 respectively from 5899 (75%) in 2013. It was only starting from the end of 2014 to the first quarter of 2015 that the outbreak reached its peak. People might have been afraid of infection and restrained from attending health facilities in the year 2015. The percentage was 74% for the year 2016 showing a significant increase after the outbreak.

Percentage HIV positive TB patients started or continued on CPT was only 8% in 2010 and reached 74% in 2013 as per the data of NLTCP of Liberia. This service was also highly affected during the EVD outbreak and decreased to 56 and 47% in 2014 and 2015 respectively. Our findings as well as the findings from similar study indicated that HIV testing among TB patients, which relies on the finger-prick test which was not recommended during an Ebola outbreak, decreased, leading to a reduction in the number of individuals diagnosed with HIV [13]. Shortcomings in ascertaining HIV status affected the uptake of both CPT needed for preventing opportunistic disease and ART [5]. Our findings were similar to the study conducted by Alyssa et al. [13].

Despite the significant impact of the 2014–2015 EVD outbreak, The NLTCP was not on hold with all TB service delivery being uninterrupted. This is the most encouraging experience during the outbreak. This was due to the commitment of the MOH of Liberia and international partners who stayed on the ground and continued their support throughout the outbreak. A study conducted in Guinea on the effect of EVD on TB program performance showed that TB program activities and performance were sustained, all TB facilities remained open, no stock out of TB drugs as result of the presence of enhanced health systems support to uphold TB services by international partners [23]. This was encouraging lesson and in stark contrast to other health settings in Guinea, Sierra Leone and Liberia where the study findings indicated negative impact on TB treatment outcome, treatment success rate and HIV testing of TB patients, TB drug stock outs, high rate of loss to follow up, facility closures and death of health workers [24, 25].

### Recommendation

The National Leprosy and Tuberculosis Control program of Liberia should conduct country wide service readiness assessment and situational analysis to improve the EVD affected TB services delivery of the country and develop strategy that will sustainably restore the program. This will enable the program to continue its momentum before the EVD outbreak and meet country specific as well as global TB program performance targets. The NLTCP should align its outbreak preparedness and restoration strategies with the Ministry of Health and other sectors as EVD and another epidemic outbreak can happen in the region as result of expanding probable virus reservoirs. The experience gained from the 2014–2015 EVD outbreak can be used to prevent and contain similar outbreaks that may happen in the region by implementing effective surveillance and preparedness strategies.

### Scope and limitations of the study

Our study only included TB case finding and TB treatment outcome data. It could have been significantly improved by including other TB program data such as the TB program financing. Besides, TB program performance data from **G**uinea and Sierra Leonne**;** the other two EVD affected countries should have been included to provide regional perspective of the outbreak.

The findings of the study clearly indicated the impact of EVD on the TB treatment outcome and laboratory diagnosis of the NLTCP of Liberia. Every single aspect of the TB treatment outcome and case finding may not be reflected on the study but the major areas that reflect the overall TB treatment outcome and laboratory performance of the program were addressed. The qualitative study interview and one of the two FGDs were conducted in the most populated city of the country, Monrovia where more than 50% of the population of Liberia lives. The other counties were not included in this part of the study though the other components of the study included the entire country. This was considered as one methodological limitation of the study. The other methodological limitation of the study was the use of focus group discussion as an instrument for the qualitative part of the study; the participants might have felt peer pressure to give similar answers which might have affected the data obtained from the discussion.

## Conclusion

Our study findings indicated that that the National Leprosy and Tuberculosis Control Program of Liberia TB service delivery and treatment outcome was drastically affected by the 2014–2015 EVD outbreak and it should device effective and sustainable restoration strategies to address this critical bottle necks, meet national and global TB program targets and effectively contain similar outbreaks that may happen in the future.

## Data Availability

The datasets used and/or analyzed during the current study are available from the corresponding author on request.

## Abbreviations

AIDS: Acquired Immuno Deficiency Syndrome
ART: Anti- Retroviral Therapy
CTP: Cotrimoxazole Preventive Therapy
EVD: Ebola Virus Disease
FGDs: Focus Group Discussions
GFTAM: Global Fund to fight TB, AIDS and Malaria
HIV: Human immunodeficiency virus
LTFU: Loss to follow up
MOH: Ministry of Health
NLTCP: National Leprosy and Tuberculosis Control Program
TB: Tuberculosis
WHO: World Health Organization
